# Ant3D—A Fisheye Multi-Camera System to Survey Narrow Spaces

**DOI:** 10.3390/s24134177

**Published:** 2024-06-27

**Authors:** Luca Perfetti, Francesco Fassi, Giorgio Vassena

**Affiliations:** 1 Department of Civil, Architectural, Environmental Engineering and Mathematics (DICATAM), Università degli Studi di Brescia, 25123 Brescia, Italy; giorgio.vassena@unibs.it; 2 Department of Architecture, Built Environment and Construction Engineering (ABC), Politecnico di Milano, 20133 Milano, Italy; francesco.fassi@polimi.it

**Keywords:** photogrammetry, multi-camera, fisheye, Ant3D, narrow spaces, tunnel

## Abstract

Although the field of geomatics has seen multiple technological advances in recent years which enabled new applications and simplified the consolidated ones, some tasks remain challenging, inefficient, and time- and cost-consuming. This is the case of accurate tridimensional surveys of narrow spaces. Static laser scanning is an accurate and reliable approach but impractical for extensive tunnel environments; on the other hand, portable laser scanning is time-effective and efficient but not very reliable without ground control constraints. This paper describes the development process of a novel image-based multi-camera system meant to solve this specific problem: delivering accurate, reliable, and efficient results. The development is illustrated from the system conceptualization and initial investigations to the design choices and requirements for accuracy. The resulting working prototype has been put to the test to verify the effectiveness of the proposed approach.

## 1. Introduction

The field of geomatics has been constantly changing and expanding due to numerous technological advances. The traditional and most consolidated surveying techniques relied primarily on the punctual recording of discrete and precise measurements requiring skilled operators and precise instruments such as levels, theodolites, tacheometers, classical aerial photogrammetry, and GNSS (Global Navigation Satellite System). In recent years the field has flourished with newer instruments and methods aimed at quickly recording a complete 3D representation, producing a dense point-wise geometric description of the object surfaces, also known as the point cloud, as is the case with terrestrial laser scanners (TLSs), portable laser scanners, airborne LiDAR (Light Detection And Ranging), and Structure from Motion (SfM) and image dense matching. These 3D dense geometric recordings of reality have enabled many new applications and are now widely adopted in fields such as land mapping, construction, cultural heritage, archaeology, and infrastructure. Moreover, software development and advancements in algorithmic processes have opened the door to non-specialized instruments as well to be used for geomatics applications with great success further expanding the field. A noticeable example is the democratization of photogrammetry thanks to modern image-based modelling software and the support for low-cost consumer-grade hardware such as DSLR (Digital Single Lens Reflex) cameras, smartphone cameras, and UAVs (Unmanned Aerial Vehicles). However, despite the many advances achieved so far, such as laser scanning and SfM photogrammetry efficiency, there are applications where these techniques cannot effectively be used due to several limitations in manoeuvrability, acquisition range, execution time, and error propagation. For example, narrow spaces, tunnels, and caves remain a challenge when accurate dense mapping is required.

Hand-carriable and backpack-mounted mobile mapping systems (MMSs) such as many commercial solutions nowadays available on the market, Geoslam Zeb Horizon [1], Leica Geosystems BLK2GO [2], Gexcel s.r.l. Heron [3], NavVis VLX [4], etc., are ideal instruments for indoor 3D mapping thanks to their manoeuvrability and speed-effectiveness of the survey operations. However, when employed in extensive or meandering narrow spaces and tunnel-like environments, the global accuracy attainable from these devices is hampered by drift error propagation [5] thus leaving the problem of efficiently digitizing narrow spaces unsolved. As an example, performing the geometric 3D survey of narrow tunnels or spiral staircases [6,7,8,9] are challenging tasks: employing a TLS is a burdensome and impractical process, even employing the newest more productive TLS solution that allows for data pre-registration on the field, such as the Leica RTC360 [2]; the field acquisition is optimized with a portable MMS; nonetheless, the unpredictable drift of the sensor’s estimated trajectory forces the practitioners to integrate the efficient MMS survey with traditional burdensome ground control measurements.

Among portable range-based MMSs, those that are practically employable in narrow spaces, such as the Geoslam Zeb Horizon [1] and other commercial instruments [10,11,12] or similar devices from the research community [13,14,15,16], cannot rely on GNSS modules, and can only house compact low-grade IMU (Inertial Measurement Unit). Thus, a refined estimate of the device’s position, movement, and trajectory is computed from algorithmic processes, i.e., SLAM (Simultaneous Localization and Mapping) algorithms [17]. SLAM methods compute the device movements in unknown environments by exploiting the 3D geometry acquired by the LiDAR mapping sensors. They are prone to failure when ambiguous or featureless geometry is supplied. Even when suitable 3D geometry is available, SLAM is prone to drift error in long acquisitions, and this error is contained if loop closures are provided during the data acquisition. However, loop closures are usually inherently denied in tunnel-like environments. Indeed, the very scenarios in which hand-carriable MMSs would be most useful are the same scenarios that tend to hamper the possibility of performing loops (tunnels, corridors). The same is true for visual SLAM methods, using image data instead of LiDAR acquisition to compute movements [18]. The visual SLAM approach works for ambiguous and featureless geometries while failing for poor image radiometric texture. The visual approach is more promising for the survey of narrow spaces and tunnel-like environments since these tend to be geometrically monotonous but rich in radiometric texture. Refs. [6,7,8,19,20,21,22,23,24,25,26] hinted that the image-based approach might be the most promising solution for the effective survey of narrow spaces, providing good robustness to drift error and good global accuracy.

### 1.1. Background

The importance of effectively computing the complete 3D survey of narrow spaces is testified by the many literature records investigating or proposing new methods to tackle this problem. Many scholars tested novel image-based techniques and novel tools alternative to the TLS in search of more time-effective and practical solutions. Among the investigated methods, there are off-line SfM and on-line visual SLAM. Promising approaches of interest specifically to tackle narrow spaces are photogrammetry employing fisheye lenses [6,7,8,9,27,28], action cameras [27,29,30,31], or 360° cameras [19,32,33,34,35,36,37,38,39,40,41,42], and custom-assembled stereo- and multi-camera prototypes [43,44,45,46], most recently coupled with on-line visual SLAM processes [20,21,47,48,49]. Fisheye photogrammetry gained attention thanks to the inclusion of fisheye’s mapping functions support in commercial SfM software, and researchers started employing it for the survey on narrow spaces, complex areas, and rapid mapping, exploiting the wide field of view to significantly reduce the number of images required.

Regarding DSLR fisheye photogrammetry, utilizing high-end cameras and professional lenses, there are fewer literature accounts: Ref. [9] proposed the use of DSLR fisheye photogrammetry to connect different image-blocks acquired for different portions of a building (inside/outside) and to survey narrow spaces, presenting the example of a spiral staircase reconstruction generated acquiring just three images per step. Ref. [8] listed the survey of spiral narrow staircases among those digitalization tasks that are challenging for TLSs and more efficient for image-based techniques, showcasing DSLR fisheye and ultra-wide lenses photogrammetry. Ref. [6] tested DSLR fisheye and ultra-wide lenses in the survey of a narrow spiral staircase acquiring five images per step employing the same image network geometry repeatedly. Ref. [27] showcased the capability of fisheye photogrammetry coupled with the commercial software Pix4D (version 1.2) by testing multiple digital cameras equipped with fisheye and ultra-wide lenses; this case study is the survey of the exterior walls and surroundings of a castle building that shows how, thanks to the wide field of view (FOV), fisheye photogrammetry can also be used as an on-the-move mapping technique potentially completing a large survey in a short time by acquiring images of a walking path. Ref. [7] presented a method to survey a narrow hypogea environment using a DSLR equipped with a 16 mm fisheye lens: the method consists of navigating the underground tunnels and acquiring a video sequence with the camera pointing forward. The camera calibration is accomplished beforehand while, in post-processing, 3 frames per second are extracted for an SfM off-line processing. The authors report promising results in terms of acquisition time and ease of operations. The accuracy is estimated in the range of a few metres (<10 m) for a tunnel length of around 1 km and the author highlights the high-tie-points multiplicity due to the high image overlap. Ref. [28] applied the same methodology to the survey of an underground cave with narrow passages and estimated an accumulated error drift during the survey path of <5 cm.

Other researchers investigated fisheye photogrammetry employing cost-effective action cameras such as the GoPro cameras, made popular for image-based modelling by the manufacturers of early UAV systems that employed them before developing custom camera systems, and that proved promising for expeditious surveying as well: Ref. [29] proposed a method to use the action camera GoPro Hero 3 for photogrammetric application, and their methods rely on obtaining an accurate calibration of the fisheye lens to then generate distortion-free perspective images prior to the SfM processing. Ref. [30] investigated different calibration procedures and checked the achievable accuracy of the action camera GoPro Hero 4 for the target scenario of the UAV survey. The rolling shutter is highlighted as the main issue for moving acquisitions; the author suggests pre-correcting the fisheye distortion and performing a camera pre-calibration of the rendered perspective images for more robust results. Ref. [31] employed the GoPro Hero 3 for the survey of a narrow corridor and the generation of orthomosaics of the side walls, and they reported centimetric-level error of Check Points (CPs) (around 3 cm). After testing both the processing of fisheye images directly with the fisheye camera model and the processing of the pre-corrected ones with the regular perspective camera model, they state that better results are achieved with the former method. Ref. [27] included a GoPro Hero 3 camera in their comparative test of fisheye photogrammetry highlighting the prominent rolling shutter effect as a limitation to accuracy and faster acquisition speed.

The majority of the literature accounts propose approaches that use panoramic 360° cameras, either ready off-the-shelf [19,32,33,36,37,38,39,40,41,42] or assembled by rigidly mounting inexpensive action cameras [34,35]: Ref. [34] proposed a method to rapidly survey indoor environments using a 360° assembly of GoPro cameras. The author investigated interior orientation calibration and the relative orientation between the action cameras and tested both still images and a video recording capturing method. Limitations were found in the synchronization of the cameras especially evident for the on-the-move video acquisition, as the author noted that post-acquisition synchronization accuracy of the video recordings is limited to the camera’s frame rate. Similarly, Ref. [35] investigated the capabilities of a 360° assembly of GoPro cameras stressing the importance of proper sensor calibration in achieving accurate 3D reconstruction. Ref. [33] tested the use of a 360° camera composed of 36 sensors (Panono 360°) in the framework of spherical photogrammetry and reported no reduction in accuracy from the traditional method of using a panoramic head while significantly speeding up the acquisition. Refs. [36,37] tested the performance of inexpensive 360° cameras, proposing a methodology for improving the quality of the equirectangular stitching. The authors highlighted the speed advantage of 360° photogrammetry stating that the method can be effective up to the representation scale of 1:100. Ref. [38] tested different 360° cameras evaluating the accuracy on control points and compared the dense image-matching result against a reference TLS point cloud, arriving at the same conclusion that the 360° camera approach is time-effective and suitable for up to a 1:100 representation scale. The authors suggested that higher metric accuracy can be achieved by investigating the distortion introduced by the sensors and the stitching algorithm. Ref. [40] tested a low-cost approach to digitizing cultural heritage using an inexpensive 360° camera acquiring pre-stitched equirectangular images. The authors concluded that even if the spherical photogrammetry can be employed successfully for immersive panoramic tours, the accuracy and noise of the resulting reconstruction were not suited for cultural heritage recording, attributing the low accuracy to the unreliable equirectangular stitching algorithm. Ref. [39] investigated the performance of two 360° cameras in the challenging task of surveying the indoor narrow spaces of a bell tower comparing the processing of single fisheye images with the processing of the stitched equirectangular images. The authors conclude that better results are achieved by processing the individual images which produced a 3D reconstruction suitable for a 1:200 representation scale with a deviation of several centimetres (around 10 cm) from the ground truth in areas not constrained by Ground Control Points (GCPs). On the other hand, they state that processing the equirectangular images resulted in higher point cloud completeness. Similarly, Ref. [19] proposed a methodology to survey underground burial chambers that uses a 360° camera fixed on a tripod. The authors compared the performance of different equirectangular stitching methods and of single fisheye images reporting better results for the latter approach yet finding the stitching method based on depth maps, estimated from the cameras’ overlapping regions, performing closely. Ref. [41] tested spherical photogrammetry for the extensive survey of the narrow street of an urban city centre performed by carrying the camera while walking. The authors state that initial estimates for the external orientation (EO) parameter were mandatory to orient the complete image block and that reliable GCPs cannot be avoided in extensive acquisition. Ref. [42] illustrated a sensor integration approach for the high-detail survey of a cloister, and 360° cameras were used to quickly acquire the narrowest areas of the cloister connecting them to a UAV image block.

Other authors, while recognizing the FOV, speed, and manoeuvrability advantages of fisheye photogrammetry and especially action cameras, instead of testing panoramic 360° configurations, investigated stereo- and multi-camera arrangements with significant baselines between the cameras. Ref. [43] experimented with a multi-camera assembly of GoPro cameras installed on a rigid bar to be mounted on a car roof to dynamically survey street tunnels, roundabouts, and roads. The authors compared different configurations of four cameras and compared the dense image-matching results with TLS point clouds reporting centimetric-level deviations (3–10 cm) with the use of GCPS along the surveyed test path. The authors do not mention the effect of rolling shutter yet stressed the importance of GCPs. Ref. [44] designed a multi-camera mapping system composed of multiple stereo pairs meant for the dynamic 3D survey of indoor spaces. They proposed a low-cost prototype based on the GitUp Git2 fisheye action cameras conceptualizing different configurations for a mobile imaging system considering both the possibility of mounting the camera closer together, allowing for a potential 360° stitching, or further apart, improving triangulation accuracy. The authors report errors in indoor (small rooms) test reconstruction of around 3 cm from the reference highlighting the main limitation of the system being the rolling shutter sensors and low-level cameras’ synchronization. Ref. [45] tested an image-based approach for railways’ tunnel inspection, proposing a multi-camera assembly comprising GoPro cameras and LED lights and reporting satisfactory results for the task at hand at a fraction of the hardware cost of a TLS approach. Ref. [46] proposed a multi-camera imaging device meant for the accurate survey of tunnels. The device, “Tunnel-CAM”, is composed of seven high-resolution DSLRs equipped with ultra-wide FOV lenses mounted on a pole in a compact and almost panoramic configuration together with powerful LED lights. The system has the advantage of recording the narrow environment at a higher resolution than most other approaches and the disadvantages of being bigger and more difficult to manoeuvre. Authors report a global error of around 20 cm on reference points in the survey of a test tunnel 300 m long. Ref. [20] proposed a novel stereo camera system meant to simplify data capture for 3D point cloud generation in urban design and historic documentation comprising two industrial cameras mounted in stereo configuration inside a compact and lightweight device to be used handheld by a single operator. The authors state that the system has the advantage of simplifying data capture since it does not require advanced knowledge about photography and photogrammetry since the acquisition is continuous and performed automatically, exploiting visual SLAM. On the other hand, the system does not mount fisheye optics and has therefore a limited field of view that can limit its applicability or ease of operations in narrow spaces. Similarly, Ref. [21] proposes a stereo camera system using industrial cameras to be used handheld for 3D point cloud generation exploiting automatic processes and visual SLAM to simplify image-based modelling for non-expert users. The authors propose a modular system where cameras and optics can change to fit specific applications such as narrow space surveys with fisheye optics [47,48].

In conclusion, the literature is rich in promising tests, prototypes, and methods to achieve the rapid survey of narrow spaces using image-based modelling. Fisheye optics are investigated as the obvious choice to contain the number of images of the photogrammetric network and to simply the capturing geometry with respect to the higher image count required using rectilinear lenses. DSLR-based fisheye photogrammetry proved effective yet requires expertise by the operator in performing the complex image capture and, moreover, because of that, still requires care and time during field operations. On the other hand, 360° photogrammetry simplifies the capturing phase further by comprising multiple viewing angles into one multi-camera, and yet the equirectangular stitching is usually unreliable, hampering the achievable accuracy; moreover, successful approaches usually require a static tripod mount. Action cameras, low-cost custom rigs, and multi-camera assembly based on action cameras perform similarly to 360° photogrammetry but are usually employed dynamically. These approaches also simplify the acquisition for non-expert users but usually at the expense of accuracy due to the camera’s instability (rolling shutter and poor synchronization). The most advanced systems take the custom multi-camera approach further, overcoming the drawbacks of the camera’s instability with more specialized hardware. Moreover, visual SLAM and other automatic processes can be included to empower the non-expert user. However, not all applications require the same hardware choices: the stereo systems proposed by [20,21] are not optimized for extensive narrow space surveys due to limited field of view and rig geometry.

### 1.2. Research and Paper Objectives

As mentioned, the most consolidated geomatics techniques are not effective for the survey of narrow spaces: both terrestrial laser scanning and DSLR close-range photogrammetry are regarded as reliable and accurate techniques; yet, in elongated tunnel-like environments, they both require acquiring a large number of data (scans or images) that usually make the job impractical; portable MMSs widely available on the market suit the task but are not regarded as reliable due to the drift error that accumulates in long unconstrained acquisitions [5]. They are accurate locally but fail in general accuracy if they are not supported by control measurements.

Complex confined areas are not uncommon, and, nowadays, acquiring these kinds of places can be necessary for many fields that would benefit from a complete 3D digitization process and extensive photographic documentation useful for restoration, inspection, and monitoring. In cultural heritage, there are narrow passages, stairways, and utility rooms; in archaeology, there are catacombs and underground burial chambers; in land surveying, there are natural formations such as caves; and in infrastructure, there are tunnels, aqueducts and sewers, or even mining. In all these types of spaces, there is a growing need to record 3D geometry, often quickly and recursively, safely, and cost-effectively.

The study described in this paper aims to provide a trustworthy and effective survey methodology for small, tunnel-like areas. Building on a prior study conducted by [6], the primary goal of this research is to leverage the robustness of SfM and comprehend drift behaviour while streamlining the process of capturing large amounts of images in a repetitive tunnel-like environment. The objective was to develop a multi-camera system equipped with fisheye lenses that can collect data quickly, intuitively, and even in the most complex and challenging spaces, producing results that are accurate and reliable enough to meet the requirements of the scale of architectural representation (2–3 cm error).

The key goals to achieve were as follows:

- Cost-effectiveness: To be competitive for low-budget applications and for the survey of secondary spaces for which laser scanning cannot be justified, such as geology and archaeology.
- Speed-effectiveness: Like the other MMSs, it must speed up the acquisition process regardless of the complexity of the space to be surveyed (narrow and meandering spaces).
- Reliability: The time saved on site must not be spent during data elaboration due to unreliable processes. This is probably the most important flaw of today’s MMSs, and it is also a problem encountered in the early tests with fisheye photogrammetry.

Therefore, the objective was to develop a multi-camera device that is compact, lightweight, and transportable by hand and houses multiple cameras to cover the entire environment in which the device is immersed except for the operator. The cameras should be equipped with fisheye lenses to maximize the field of view and minimize the number of images to be acquired to complete the survey. The compact structure should accommodate the cameras by ensuring a robust fixed baseline between all cameras in the system. The constrained fix design will then allow for automatic scaling of the resulting three-dimensional reconstructions, introducing the relative orientation constraints between cameras and reducing the degrees of freedom of the photogrammetric network.

This paper describes the research that led to the design of a working prototype of a novel instrument, a fisheye multi-camera called Ant3D that resulted in a patent application in 2020 and that has already been tested and compared multiple times in the field against other approaches [5,22,23,24,25,49,50].

### 1.3. The Beginning of the Research—The FINE Benchmark Experience

**Figure 1:**
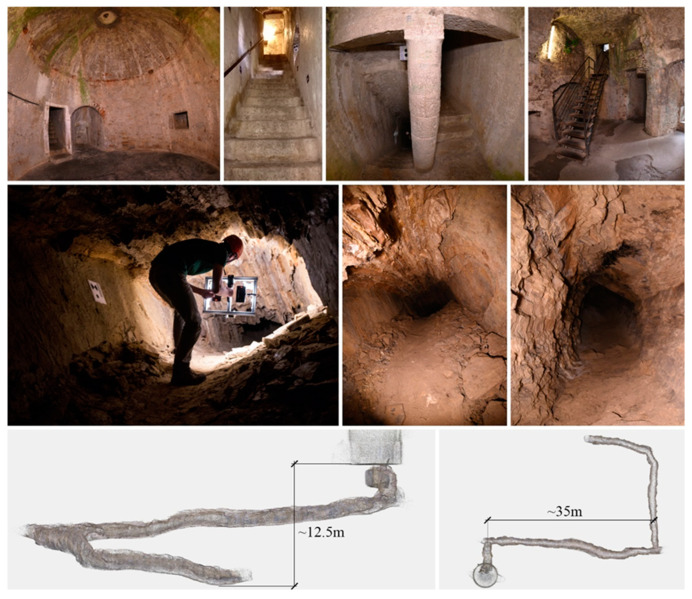
Some images of the San Vigilio Castle’s tower (**top**) and tunnel (**centre**); and the dimensions of the tunnel (**bottom**). The image refers to the FINE benchmark dataset.

At the beginning of the research interest in fisheye photogrammetry and fisheye multi-camera applications, in 2019, an access-free benchmark dataset was designed to provide a set of data to evaluate the performances of different image-based processing methods when surveying complex spaces, specifically the performance of low-cost multi-camera rigs: the FINE benchmark (Fisheye/Indoor/Narrow spaces/Evaluation). Participants from academia and research institutes were invited to use the benchmark data and demonstrate their tools, codes, and processing methods in elaborating two image datasets for the 3D reconstruction of narrow spaces (Figure 1). The benchmark dataset was first presented during the 3D-ARCH 2019 conferences held in Bergamo, where a special session was held specifically for the presentation dealing with the benchmark.

The benchmark data were acquired in the internal spaces of the Castagneta Tower of San Vigilio Castle, located at the very top of Città Alta (Bergamo, Italy). The case study has been chosen because of the co-existence of challenging conditions that can be exploited to stress the techniques and processing strategies. All the indoor spaces of the castle are poorly illuminated, and the two main environments of the tower include some narrow passages in the range of 70–80 cm wide. They differ in the surface features: artificial, refined flat surfaces for one area and rough natural rock surfaces for the other.

The benchmark was composed of two datasets referring to the two connected environments:

- Tunnel: a dark underground tunnel (around 80 m long) excavated in the rock, with a muddy floor and humid walls. In some areas, the ceiling is lower than 1.5 m.
- Tower: an artificial passage composed of two rooms with a circular/semi-spherical shape that are connected by an interior path, starting from the tower’s ground floor and leading to the castle’s upper part, constituting staircases, planar surfaces, sharp edges, walls with squared rock blocks, and relatively uniform texture.

**Figure 2:**
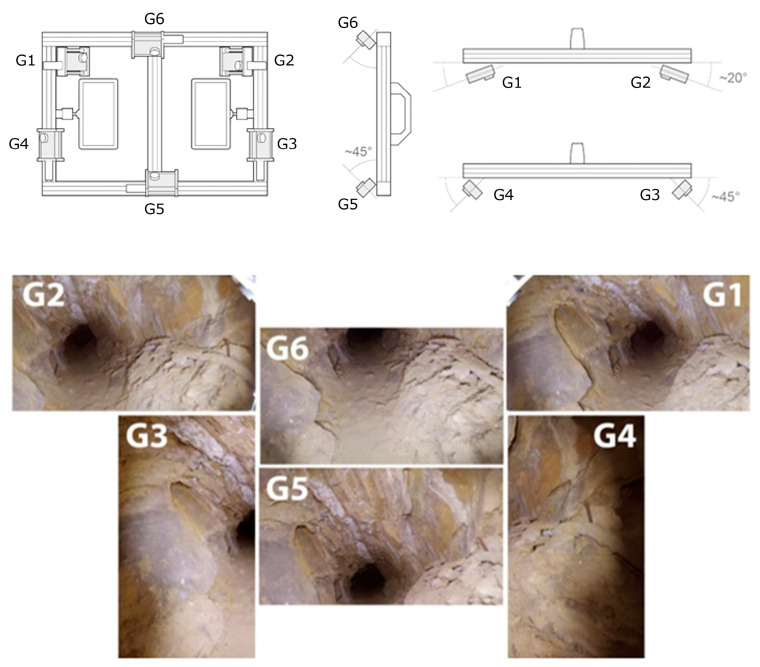
The GoPro array scheme (**top**) and an example of the 6 views inside the underground tunnel (**bottom**). The image refers to the FINE benchmark dataset.

The FINE benchmark provided several data including the image datasets and a laser scanner ground truth point cloud. For the acquisition of the low-cost multi-camera datasets, an array of action cameras was used to perform a rapid video acquisition of both the tunnel and tower areas. The rig consists of six GoPro cameras mounted rigidly on a rectangular aluminium structure (Figure 2). Continuous light is provided by two LED illuminators mounted on the back.

The rig was designed to have a sufficient base distance between the six cameras in relation to the width of the narrow passages. The design was thought to reconstruct the object geometry at every single position of the rig. Two cameras were mounted on the top (G6) and the bottom (G5) of the structure, tilted roughly 45° degrees downwards and upwards. Four cameras were mounted on the rig’s sides, two of them (G1, G2) in a convergent manner oriented horizontally, and two in a divergent way (G3, G4) oriented vertically.

**Figure 3:**
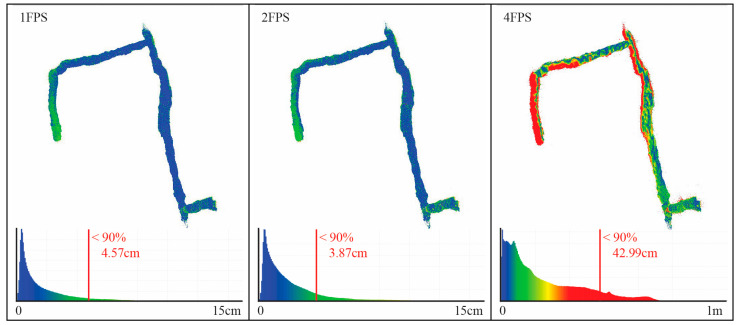
Cloud-to-cloud validation of the point clouds against ground truth. The maximum deviation error considering the better 90% of the points reported. The image refers to the FINE benchmark dataset.

The FINE benchmark provided the basis for an in-depth test of the low-cost multi-camera approach. Our own investigations comprise the synchronization of the individual video sequences of the six GoPro cameras using the audio tracks available and the subsequent extraction of timestamped keyframes to form the image datasets to be used for SfM. The obtained images were then processed using a pipeline implemented with the commercial software Agisoft Metashape (version 1.7) that accounts for rigid constraints of the known baselines between the cameras. Different keyframe extraction densities were tested, namely 1 fps, 2 fps, and 4 fps. The evaluation of the resulting 3D reconstruction of the processed datasets was performed in two ways: (i) by checking the error on CPs available along the narrow environments and extracted from the ground truth laser scanner point cloud, and (ii) by checking the cloud-to-cloud deviation of the obtained sparse point cloud from the reference ground truth. For both evaluations, the multi-camera reconstructions were oriented with the reference point cloud using a few GCPs at the tunnel start in order to check the maximum drift at the opposite end. Table 1 shows the error on the checkpoints resulting from the 3D reconstruction of the tunnel environments for the 1, 2, and 4 fps datasets. Figure 3 shows their relative cloud-to-cloud deviation from the laser scanner ground truth point cloud. For all results reported, the baselines between the cameras were rigidly constrained in the bundle adjustments exploiting the scalebar function available in Metashape. Overall, the error obtained exceeded the target accuracy, and the processing presented a great degree of unreliability.

The FINE benchmark’s experience revealed several problems with the multi-camera implementation based on a commercial action camera. Nevertheless, the results confirmed the potential of the image-based multi-camera approach, allowing for the complete acquisition in a short time despite the complexity of the environment. However, reaching architectural accuracy (2–3 cm) was impossible without using the coordinates of known points measured with the total station to optimize the three-dimensional reconstruction.

The main limitations were as follows:

- The geometry of the multi-camera. The used configuration, consisting of six GoPro cameras oriented mainly in the frontal direction combined with the surface roughness of the rock walls, has resulted in an insufficient number of tie points to connect the images acquired in the forward direction with those obtained in the backward direction.
- The rolling shutter of the sensors used. The introduction of distortions due to the acquisition in motion and the use of rolling shutter sensors has led to not being able to accurately calculate the camera’s internal orientation parameters and not being able to impose constraints on the relative orientation of the cameras without high uncertainty. Nevertheless, the constraints on the distances between the cameras were effective in reducing the drift error compared to non-constrained processing.

The FINE benchmark experience highlighted how, in order to achieve the aforementioned goals, a custom system was necessary to overcome the low-cost hardware limitation. The chapters below describe the hardware and design choices that led to the definition of the current system.

## 2. Materials and Methods

In designing the multi-camera system, the analysis considered together both the fisheye mapping function characteristics specific to the hardware in use and the environmental characteristics of the targeted applications for the system. The proposed multi-camera design was optimized for the following target environments, which is thought to be a good approximation of common narrow spaces: a tunnel measuring 1 m width by 2 m height. The main topics tackled are (i) the multi-camera stability with movements, (ii) the multi-camera calibration of both interior orientations and relative orientations of the cameras, and (iii) the design of the multi-camera arrangements, i.e., the optimal rig geometry for the multi-camera-system derived through a study on the GSD (Ground Sampling Distance) distribution in object space accomplished through simulation.

### 2.1. Materials

The hardware used throughout the investigation and for the proposed prototype is composed of 5 industrial-grade RGB cameras: the FLIR BlackFly S U3-50S5-C that uses a global shutter 5-megapixel 2/3″ colour sensor with a pixel pitch of 3.45 μm. Each camera is equipped with a 190° circular fisheye lens SUNEX PN DSL315, an equidistant fisheye with a focal length of 2.7 mm, and an FOV of 190° in an image circle of 7.2 mm. The lens was chosen so that the image circle would fit the sensor, keeping almost the entirety of the field of view. As highlighted from the FINE benchmark experience, the global shutter sensors and the possibility of accurately triggering the shots were needed to guarantee multi-camera stability in the presence of movement. The 5-megapixel resolution was chosen based on the narrow nature of the target environment, not requiring high-resolution images to grant acceptable GSD, and based on the need to contain computation effort in processing a high number of images.

### 2.2. Multi-Camera Stability with Movements

In designing the improved multi-camera rig, the first problem addressed was the lack of frame synchronization experienced in the previous tests with the action cameras. The problem has been tackled by defining a maximum threshold for the displacement error of a given object point in image space due to the synchronization error and the presence of relative motion between the object and the camera rig. This threshold has been set to the size of 1 pixel so that it would not be detectable in the images. Then, also considering the effect of the fisheye mapping function, the maximum synchronization error that would generate a displacement of 1 pixel in image space in the operational conditions (movement speed: 1 m/s, camera-to-point minimum distance: 1 m) is derived.

Then, the actual synch error of the multi-camera system was measured with the aid of a synchronometer that can measure synch error up to 10 μs. The device works by emitting intermittent light pulses precisely spaced; by acquiring a sequence of multi-images of the device, it is possible to read out eventual delays in the camera captures. The synchronization test is passed if the multi-camera asynchrony is lower than the computed maximum synchronization error.

The same framework also applies to the computation of the minimum exposure time for the cameras so that no motion blur can be detected in the presence of relative movements between the subject and the rig. As for the maximum synchronization error, the minimum exposure time is the exposure time that causes a displacement of scene points in image space to the threshold value of 1 pixel in the target operational condition, and it also considers the effect of the fisheye mapping function.

Section 3.1 provides a framework for computing the maximum synch error and minimum exposure time considering the fisheye mapping function, together with the results of the synchronization error measurement.

### 2.3. Multi-Camera IO and RO Calibration Method

The stability of the global shutter sensors, together with accurate frame synchronization, allows for the accurate and reliable computation of the interior orientation (IO) parameters for each camera and the relative orientations of the secondary cameras with respect to the primary.

**Figure 4:**
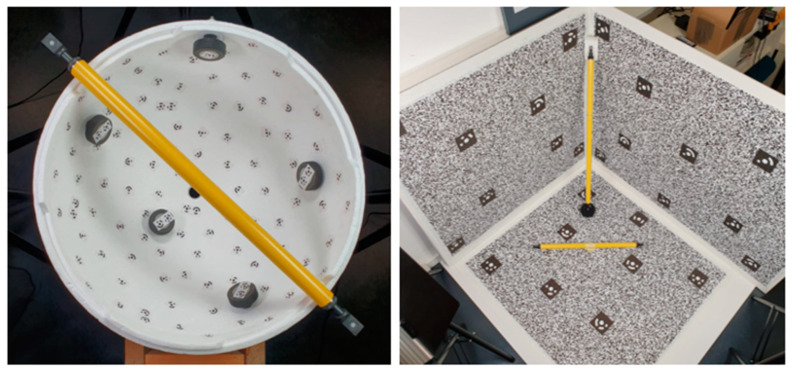
Calibration text fields: texture-less semi-sphere (**left**) and textured corner (**right**).

The IO calibration will be performed for each camera composing the rig. To achieve a reliable calibration, two different calibration test fields were compared with the aim of defining the ideal one: the first one is a texture-less semi-sphere covered in coded targets (Figure 4—left) as used by [30], while the second one is a corner-shaped highly textured test field (Figure 4—right). For both the test fields, reference 3D coordinates of some markers were measured by conducting a monocular photogrammetric acquisition with a DSLR scaled using reference invar bars. The monocular photogrammetric processing yielded the reference coordinates for both test field markers to an accuracy of around 0.2 mm (accuracy of the reference bars). Later, these coordinates were used to scale the calibration acquisitions performed with the fisheye cameras and control the result. For the comparison of the two test fields, a calibration was performed for just one camera before conducting the calibration for all cameras with the best-performing method. The fisheye calibration acquisition was performed by rotating the camera around the test field and by rotating it in all different directions. For the semi-spherical test field, the camera was roughly positioned along the imaginary other half of the semi-sphere pointing to the test field with the optical axis pointed to the centre of the sphere; during the acquisition, the camera was also rotated around its optical axis (roll). Roughly the same approach was followed for the corner-shaped test field by moving the camera over an imaginary semi-sphere.

Other than the internal orientation parameters, the camera’s relative orientations can also be calibrated and used as constraints during the system deployment to reduce the degree of freedom of the photogrammetric network. The relative orientation relationship between the cameras allows us to constrain the baselines between them as well as their rotations.

**Figure 5:**
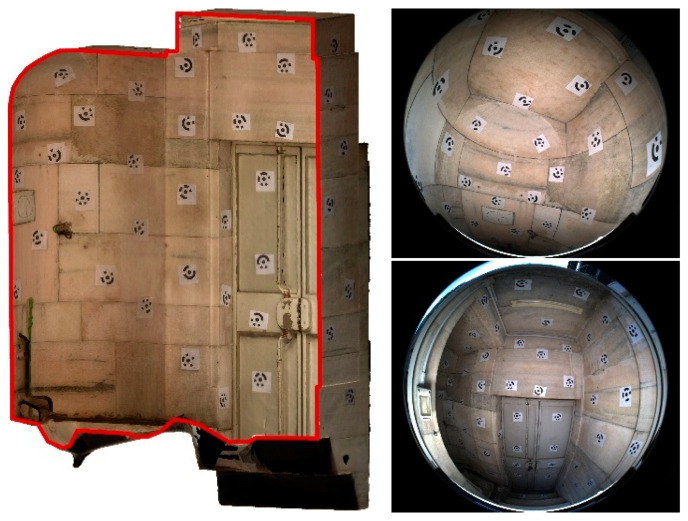
A section view of an example calibration test field set-up on the field for the self-calibration of the multi-camera baselines (**left**) and two images acquired by the multi-camera system (**right**).

The RO calibration process is performed using Agisoft Metashape in a pipeline like the one already described for the single cameras’ calibrations. That is, by performing a photogrammetric acquisition with the assembled multi-camera system of a known test field that is preferably a small “room” of similar dimensions as the size of the environment, the system is intended to be used (Figure 5). The multi-camera acquisition is then processed to the best possible orientation of the image network. From the estimated coordinates of the oriented cameras, the calibrated RO is computed through Metashape. Reference coordinates for the targets are used half as a constraint to scale the reconstruction and half as a check.

### 2.4. Designing the Multi-Camera Arrangement

The study to define the multi-camera rig geometry that is improved from the rig geometry used in the FINE benchmark is firstly based on some practical considerations and lessons learned. Subsequently, pre-defined geometries that have been considered reasonable are compared based on their GSD performance, considering, therefore, the relationship between the camera’s angles and environment geometry for which the multi-camera is intended. In this test, the environment is defined as a synthetic dataset of points equally spaced on the surface of a tunnel-shaped parallelepiped of cross-section w: 1 m × h: 2 m. The synthetic dataset of 3D points is used to simulate the projection of each point onto the image plane of one or more simulated cameras. The simulated cameras can be modified both in their internal properties, e.g., principal distance and mapping function, and in their external orientation.

Initial considerations to define the reasonable arrangements to compare were (i) the rig dimensions and (ii) the choice of avoiding framing the operator and the light sources within the FOV of the cameras. On the lesson-learned side, the FINE benchmark highlighted the importance of connecting the outward acquisition with the return acquisition, and this requires planning the incidence angle of the camera’s optical axis to the surface walls. It is important that the images from the two directions of acquisition framing the same area on the walls of the tunnel are not too different, especially in the scenario of rough surfaces.

**Figure 6:**
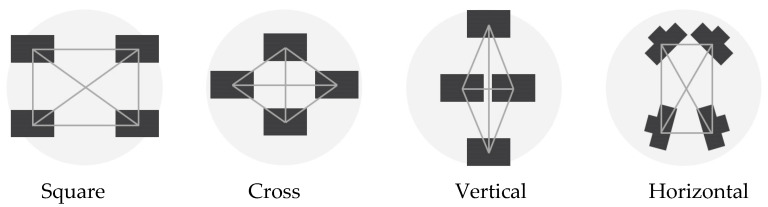
The figure reports the four families that were considered for arrangements. For each category, different orientations of the cameras were simulated and compared. All schemes depict the configuration in the vertical plane of the tunnel cross-section except for the horizontal rig, which is shown in plan view.

These initial thoughts led to the definition of the reasonable multi-camera configurations represented in Figure 6. These arrangements depict only four cameras since it has already been decided that one camera will point straight ahead in the final assembly. This choice depends mainly on the idea of exploiting the central front-facing camera in the future to run real-time processing of the data using only this camera and on the idea of using this camera for virtual inspection purposes. Therefore, only the arrangements of the remaining four cameras remain to be defined.

The reasonable arrangements are the following:

- Square: It consists of four cameras organized in two horizontal couples on top of each other. The cameras can be rotated at different angles along their vertical axis. The distance between the cameras is 20 cm, both on the horizontal and on the vertical direction.
- Cross: The cross geometry consists of a vertical pair of cameras and a horizontal pair of cameras. The cameras can converge or diverge towards the centre at different angles. As in the previous configuration, the distance between the cameras is 20 cm in both horizontal and vertical directions.
- Vertical: This geometry consists of the first pair of cameras in the vertical direction with a long baseline (40 cm) converging toward the centre at different angles and a second couple in the horizontal direction with a short baseline (10 cm) diverging and pointing toward the sides.
- Horizontal: The horizontal configuration consists of a couple of frontal cameras and a couple of rear cameras. The cameras within the two couples are close together (10 cm apart), while the front and rear cameras are positioned 20 cm apart. The longest baseline is, therefore, oriented along the tunnel extension.

As mentioned before, a fifth camera is always positioned in the centre, pointing forward for all the arrangements.

The pre-defined arrangements were then tested by simulating virtual cameras inside the synthetic dataset of the tunnel mentioned above. For each point in the synthetic dataset, a projection in image space can be simulated for each camera of the tested rigs, and different mapping functions can also be used. The GSD can be computed for each point in the 3D virtual scene for each camera so that each synthetic point holds a reference to the computed values from all cameras in which it is visible. From this, a GSD distribution analysis is performed considering the average GSD obtained for each point in the scene. This analysis allows us to draw some consideration on the tested configuration and, therefore, can help to decide which one would be the best performing in general or according to specific objectives.

The multi-camera rig geometry and the relationship between fisheye projection and incidence angle with the collimated points were considered to simulate the GSD behaviour. The GSD is therefore computed according to Equation (1) and is expressed as a function of (i) the principal distance, (ii) the distance between the camera and its normal plane passing through the point, (iii) the detector pitch, and (iv) the lens mapping function.

(1) $$GSD=h\cdot \left[\tan\left(f\left(c,r_{2}\right)\right)-\tan\left(f\left(c,r_{1}\right)\right)\right]$$

**Figure 7:**
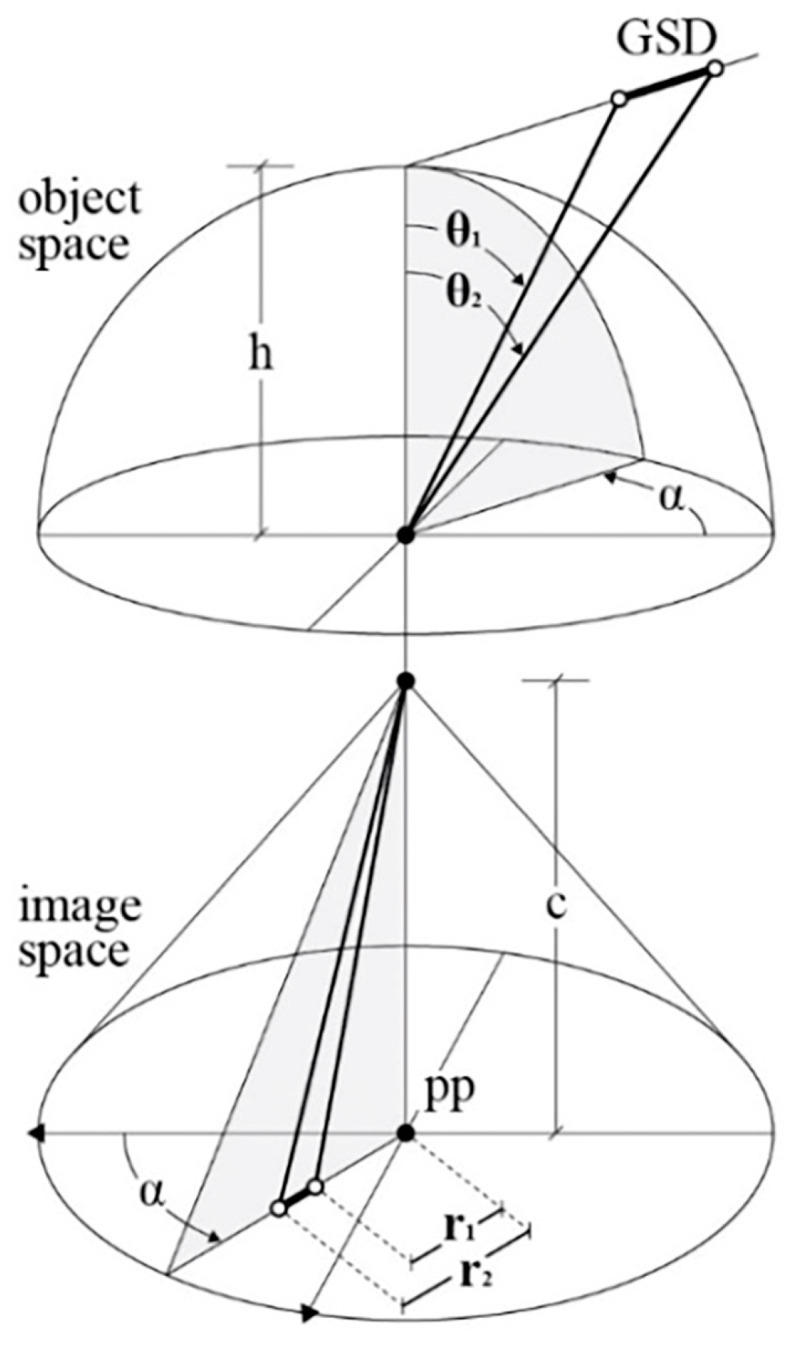
Fisheye mapping function scheme; radius from projection centre is $r=f\left(c,\;\theta \right)$, where f is lens mapping function. For equidistant fisheye projection, $f\left(c,\;\theta \right)=c\cdot \;\theta$ [51].

The notation refers to Figure 7. For each point projected onto the image plane, the radius $r_{1}$ is known, and the radius $r_{2}$ depends on the detector pitch, with

$$r_{2}=r_{1}+pixel\;size.$$

$f\left(c,\;r_{i}\right)=\theta_{i}$ is the inverse of the lens mapping function $r=f\left(c,\;\theta \right)$.

**Figure 8:**
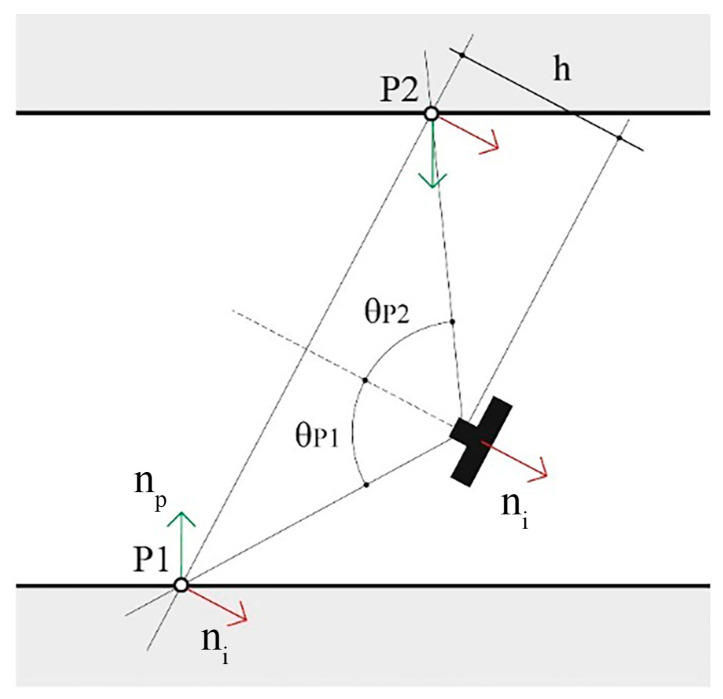
The figure illustrates the framing of two points, P1 and P2, at the same distance, h, from the camera and forming the same angle θ with the optical axis. It follows that the GSD for the two points is the same. However, the $GSD_{pW}$ is different, and the weighted metric is worse for point P1 based on the greater angle between the point normal (green arrow) and the image normal (red arrow).

Equation (1) is used to compute the GSD for each point projected onto the simulated images, the value is then stored back inside the 3D point entity. At this point two metrics are computed for all 3D points: (i) the first one is $GSD_{p}$, referring to the average of GSD values, according to Equation (1), calculated from all cameras in which the point is visible; (ii) the second one is $GSD_{pW}$, where the average of the individual GSD values calculated for each insisting camera is weighted based on the angle between the image normal and the point normal. The weights are calculated as $W_{i}=f\left(\tau \right)$, where $\tau =\cos^{-1}\left(\hat{n_{\;i}}\cdot \hat{n_{p}}\right)$, and the angle between the image and the point unit normal vectors are $\hat{n_{\;i}}$ and $\hat{n_{p}}$. The image unit normal vector $\hat{n_{\;i}}$ points opposite the viewing direction, in the direction from the optical centre to the image projection (the negative). It follows that when the camera is oriented along the point normal, looking at the point, the angle τ is 0°; conversely, when the camera is looking away from the point, the angle τ is 180° (Figure 8). $W_{i}$ maps the angle between the vectors from the range [0°, 150°] to the interval [1, 10] linearly, and for angles greater than 150°, the GSD value is discarded. The $W_{i}$ is the attempt to consider the visibility of the point as a factor in the metric; if a point lies on a surface almost occluded, that is, looking away from the camera viewing direction, it is assumed that the accuracy of point detection would be lower than the accuracy for a second point that lies on a surface that is looking towards the camera viewing direction, even if the two points forms an identical angle with the optical axis (Figure 8). The mapping of the angles to the arbitrary range [1, 10] has the effect of maintaining the GSD value unaltered in the best condition and worsening it up to ten times in the worst condition. All analyses based on this second metric have the objective of discriminating between the different camera arrangements to help decide the best ones based also on the assumption that a greater angle between the image and point normal vectors corresponds to a worse measurement quality during actual system deployment.

To find the best-performing configurations within each category/family (Figure 6), different camera orientation angles were simulated, and the results, in terms of the two metrics defined above, were compared within the same category/family. Out of each of the four considered categories, one specific best-performing configuration is therefore defined. Finally, the best-performing arrangements of each category are compared among themselves, and results are drawn from them.

## 3. Results

### 3.1. A Framework for Computing Displacement Error with Movements in Fisheye Cameras

Equations (2) and (3) give the point displacement in image space $∆s^{\prime }$ considering a general mapping function: $r=f\left(c,\;\theta \right)$ (Figure 7). Different mapping functions will produce different displacement errors in image space from the same amount of relative motion. The mapping functions $r=f\left(c,\;\theta \right)$ of the most common fisheye projections can be found at [51].

(2) $$\theta_{t1}=\theta_{t0}+arctan\frac{∆S}{D}$$

(3) $$∆s^{\prime }=f\left(c,\theta_{t1}\;\right)-f\left(c,\theta_{t0}\;\right)$$

**Figure 9:**
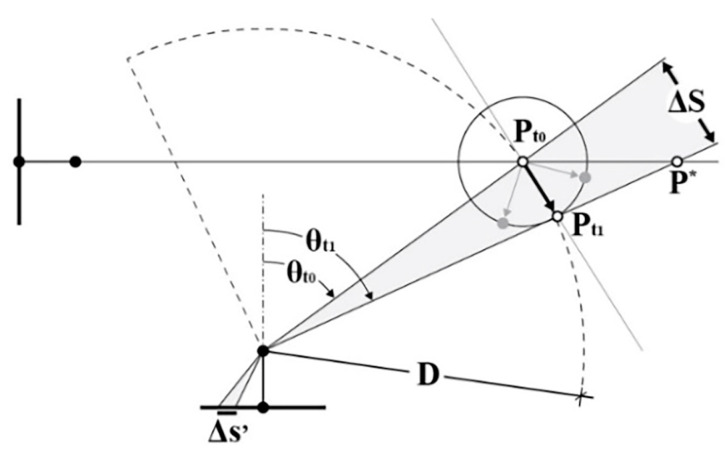
Point displacement in image ($∆s^{\prime }$) and object (∆S) coordinates due to synch error and relative movement considering fisheye projection. Adapted from [51] to take fisheye image projection into account.

**Figure 10:**
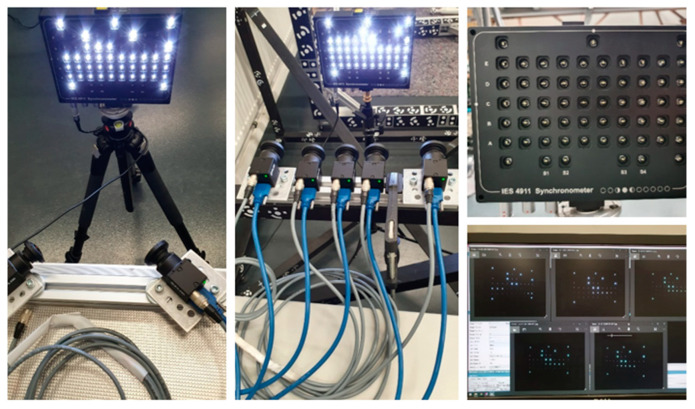
A check of the synchronization error performed with the aid of a synchronometer; the resolution of the devices is 10 μs.

With a regular slow walking speed of 1 m/s and a distance (D) of 1 m (taking into consideration the main application for the developed system), the estimated maximum acceptable synch error is ~1 ms to meet the condition $∆{s^{\prime }}_{equidistant\;fisheye}\leq pixel\;size$ (Figure 9). Firstly, a software synchronization between the cameras was tested, resulting in a measured synch error of ~30 ms, vastly surpassing the maximum level of accepted synch error. Because of that, hardware synchronization between the cameras was mandatory to meet and surpass the requirements with ~200 μs of max delay (Figure 10).

### 3.2. Multi-Camera IO and RO Calibration Results

Regarding the IO calibration comparison test, a visual inspection of the marker reprojection favoured the corner-shaped approach. For the semi-sphere dataset, displacements can be observed in the corners area. Only little differences could be observed in the evaluation of the reprojection error of control points in image space.

Based on the result of the comparison between the two test fields, the textured corner-shaped test field was used to obtain the calibration of all the cameras of the multi-camera rig. Overall, the calibrations present similar results. The correlation matrices, as expected, show a strong correlation between the radial distortion parameters and between x0 and p1 and y0 and p2. Moreover, on average, the RMSE on the markers, where all markers are used as CPs, is below 0.3 mm, comparable with the accuracy of the reference coordinates of 0.2 mm; the RMSE in image space is instead, on average, below 0.2 pixels.

Regarding the RO calibration, considering the current implementation of the prototypes and the stability of the current system, it is preferred to repeat the RO calibration process often, ideally for every deployment of the system, similarly to a self-calibration of the internal orientations. During SfM processing, the estimated RO parameters are imposed in Metashape using the multi-camera function. In previous deployments of the system, only the baselines would be estimated from calibration; in that case, the Metashape scalebar function would be used to impose the constraint, relying on a Python script that implements all baselines automatically from an input source file.

### 3.3. The Geometric Configuration of the Multi-Camera

**Figure 11:**
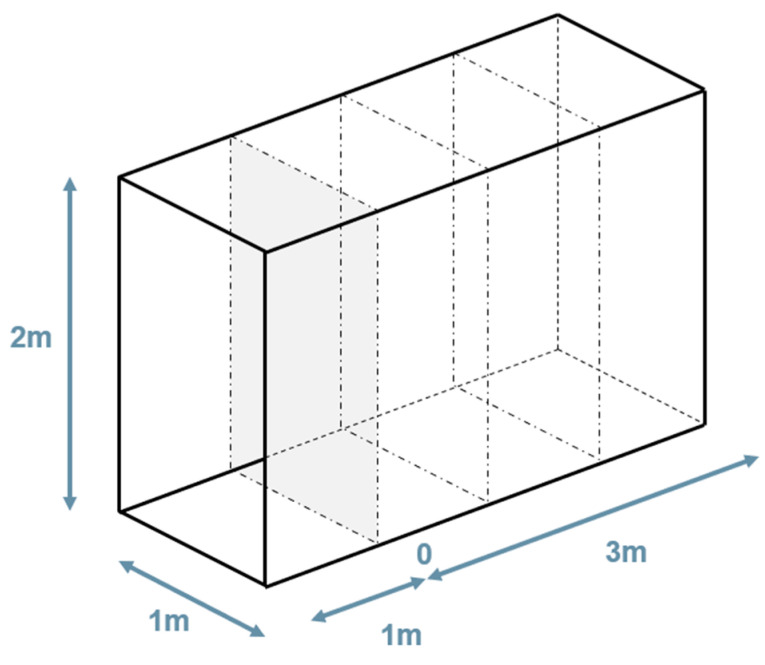
The geometry of the simulated synthetic 3D environment used during the tests; the multi-camera rigs are positioned in 0 in the centre and pointing forward.

The simulation of the GSD distribution is performed in image space for each camera in the rigs, computing the GSD according to Equation (1) for each synthetic point projected onto the simulated image. Then, the analysis is transposed in object space by computing the two metrics described in Section 2.4. Figure 11 illustrates the object space synthetic data geometry that is considered during the test. The origin of the multi-camera rig is positioned at the centre of the grey cross-section plane and pointing straight ahead along the tunnel extension.

**Figure 12:**
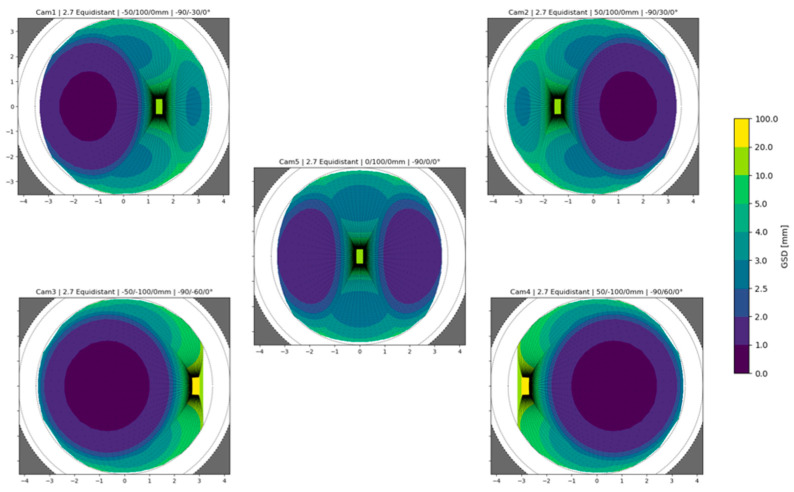
GSD distributions simulated in image space for the “horizontal” configuration with front cameras at 30° and rear cameras at 60° left and right from the front-facing direction.

**Figure 13:**
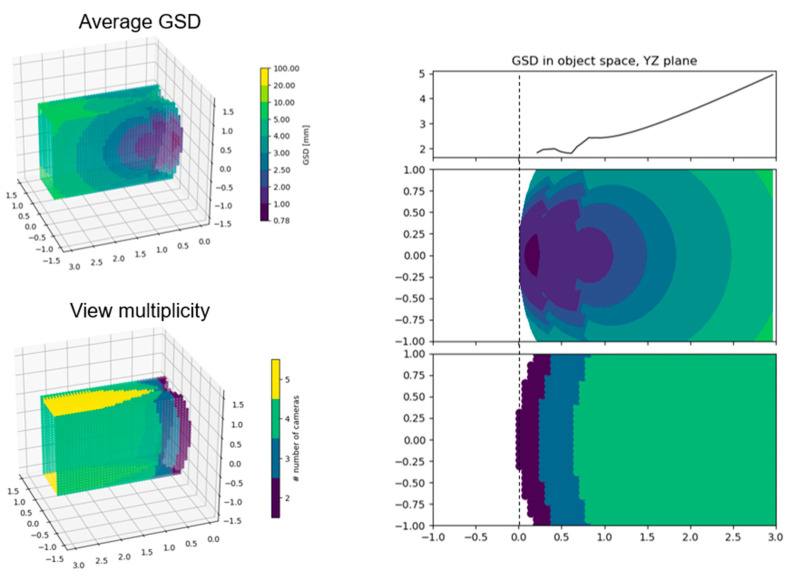
GSD distribution in object space ($GSD_{p}$ metric). The 3D plots on the left show the metric (**top left**) and the view multiplicity for each point (**bottom left**). On the right (**centre right** and **bottom right**), the same information is shown in 2D on the YZ plane of the side wall of the simulated environment. Finally, the plot at the top right shows the mean computed column-wise value of the GSD distribution on the YZ plane (**centre right**), and the curve represents how the GSD is distributed on the side wall of the tunnel. The images refer to the same “horizontal” configuration of Figure 12.

Figure 12 and Figure 13 show the plots computed for all configurations showcasing the “horizontal” configuration with camera angles set at 30° for the front cameras and 60° for the rear cameras. First, the GSD distribution simulated in image space for all the cameras composing the rig is shown; second, the object space average of these contributions is shown. In Figure 12, the image projections are cropped at 150° of the angle of incidence θ to discard the worst areas of the GSD behaviour.

Figure 13 illustrates how, from the GSD distribution in object space, a single curve is computed to represent the GSD variation on the YZ plane of the simulated tunnel (the side surface). This curve (Figure 13, top right) is computed as the column-wise mean of the plot below (Figure 13, centre right), which is the 2D representation of the GSD distribution on the side wall of the tunnel. This same procedure is repeated for both the $GSD_{p}$ and the $GSD_{pW}$ metric. Moreover, it is also repeated for the XY plane of the tunnel, obtaining a similar curve for the two metrics representing the GSD distribution variation on the horizontal planes of the tunnel (ground or ceiling).

**Figure 14:**
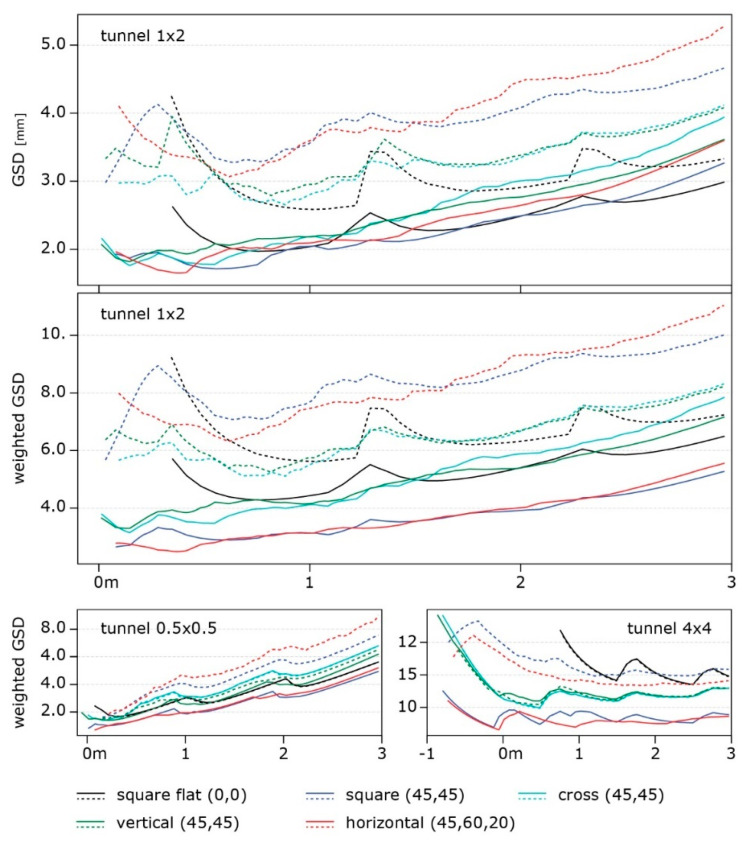
The graphs report the comparison of the selected configurations. The *x*-axis represents the length of the tunnel along its extension; the continuous and dashed lines describe the GSD distribution on the vertical and horizontal plane of the tunnel, respectively. Three consecutive poses of the multi-camera rig are considered at positions 0, 1, and 2 m. The top and centre graphs are considered virtual tunnels of 1 m × 2 m, while the bottom-left and bottom-right graphs are considered square tunnels of 0.5 m and 4 m, respectively.

Figure 14 illustrates the final comparison between the best-performing arrangements for each of the different rig families (Figure 6), each selected by choosing the best performing of all variations with different camera rotations within each family. For this plot, three consecutive poses of the multi-camera rigs are considered at positions 0, 1, and 2 m along the simulated environment (Figure 11). This is performed with the purpose of also evaluating the interaction of different poses during acquisition. Discontinuities in the GSD distribution, as it is visible for the “square flat” configuration, show inhomogeneity in the resolution at which the surfaces of the tunnel are framed.

In Figure 14, the graphs on top and in the centre show the results with the two metrics: it can be noticed (i) how the square geometry with all the cameras oriented forward (black line) covers significantly less area at the beginning of the tunnel; (ii) a clear separation of the tested geometries in two groups, square with horizontal and cross with vertical, with the former group performing the best on the side walls (continuous lines in the graphs) and much worse on the horizontal surfaces (dashed lines), and the latter group presents more balanced performances; and finally (iii), as anticipated above, the rig with the cameras pointing forward shows high peaks at each pose due to the decaying resolution on the edges of the frame, suggesting that a shorter baseline between the poses should be considered. Again, in Figure 14, two more graphs are reported at the bottom; they represent the GSD-weighted behaviour computed for different tunnel cross-sections: (i) a 50 cm × 50 cm tunnel (bottom left) and (ii) a 4 m × 4 m tunnel (bottom right). These bottom graphs highlight how a wider tunnel diameter results in a more even average resolution along the 3 m length and in amplified differences among the compared rigs.

The results allow for a distinction of different rigs in different categories that can be employed according to the specific needs of each application, such as the required resolution and the relevance of the horizontal or vertical surfaces. The “horizontal” configuration, the red line in Figure 14, performed the best on the side surfaces and entails some crucial advantages like its reduced section size compared to the length, which is ideal for inspecting small niches. Moreover, the field of view of the cameras is ideal to avoid framing the rest of the structure as much as possible.

## 4. Proposed Multi-Camera System—Ant3D

### 4.1. Description and Main Features

The study of improving the hardware of the initial multi-camera system led to the design of a working prototype of a multi-camera photogrammetric system designed for the survey of complex and narrow areas called Ant3D. The proposed device aims to be an alternative to modern dynamic 3D surveying systems on the market, offering high measurement accuracy combined with the acquisition of high-resolution images, optimal characteristics for digitization, and detailed inspection of surfaces and meandering spaces.

The device allows for a drift error reduction in long acquisitions. This is due to the combined exploitation of (i) the fisheye lens angle of view, (ii) the five cameras arranged in such a way that the whole scene except the operator is always captured in its entirety, (iii) the accurate synchronization of the captured images, (iv) the accurate calibration of the multi-camera IO and RO, and (v) the data processing through Structure from Motion and potentially through V-SLAM.

**Figure 15:**
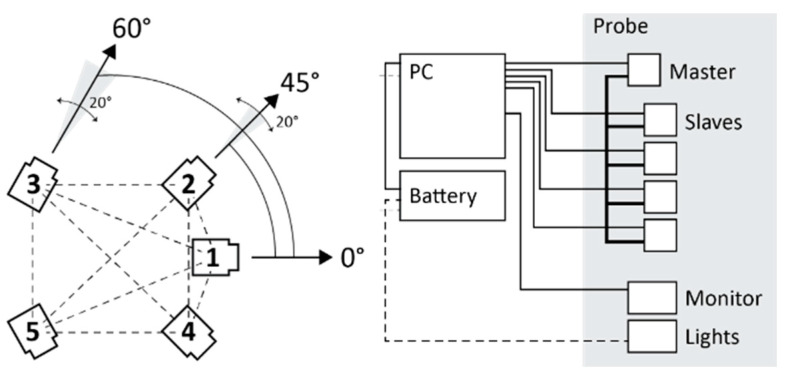
A scheme of the proposed multi-camera system. The figure reports the actual angles between the cameras used in the implementation of the prototypes and also includes a range of adjustments that could be implemented for future builds to adapt the device for different target environments. Additional sensors, such as IMUs, are not yet implemented but could be included in future iterations.

**Figure 16:**
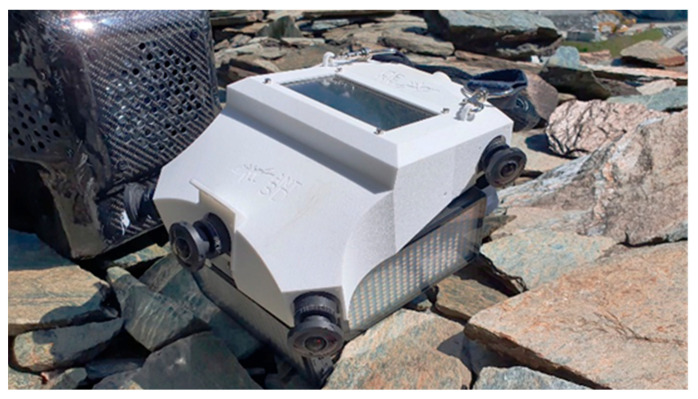
A picture of the second version of the prototyped instrument. The picture shows the probe in the front, the backpack that houses the PC, and the battery on the back on the left.

The multi-camera system is composed of two parts: a hand-held structure or probe and a small backpack connected by data transmission cables (Figure 15). The mechanical structure houses five cameras, a touch screen, and three LED illuminators (2000 lumens each). The geometry of the multi-camera is composed as follows: five cameras are configured in such a way that with respect to a horizontal plane and a front aiming direction, the first frontal camera is placed at 0° angle with respect to the aiming direction and at around 10° up with respect to the horizontal plane; the second and third front right and left cameras are placed at an angle of +45° and −45°, respectively, in relation to the aiming direction; and the fourth and fifth rear right and left cameras are placed at an angle of +60° and −60°, respectively, in relation to the aiming direction. There are about 15 cm between the right and left front cameras, and about 20 cm between the front and rear cameras. This arrangement is motivated by the study described in Section 3.3, where the horizontal configuration was the best compromise between performance and practical considerations. This configuration is designed for tunnel-shaped environments with a cross-section varying from about 1 m to about 4 m. However, the structure can also be used in narrower environments, such as niches from about 30 cm wide or even larger environments, provided that it is possible to illuminate these environments with different solutions aside from the lights mounted on the structure and accept a lower accuracy. The relative arrangement between the cameras, and particularly the presence of a significant distance between their centres, especially along the forward/walking direction, allows for an accurate triangulation of homologous points and scaling of the resulting three-dimensional reconstruction. The LED illuminators are mounted both on the side and on the front of the system and are as large as the compact dimension of the prototype allows for (around 10 × 15 cm). The current configuration has been designed to maximize the illuminator’s surface area in order to contain hard shadows and inhomogeneous illumination that is inherent in the single-point light source configuration. Figure 15 shows a scheme of the proposed system and Figure 16 displays a picture of the second prototype build based on this scheme.

The five cameras composing the system are the same type with identical fisheye lenses as detailed in Section 2.1. The 190° FOV optics allow us to reduce the number of images needed for the 360° 3D reconstruction allowing us to obtain complete coverage of the framed scene, except for the operator, using only five cameras. In addition, the wide angle of view of the multi-camera allows for obtaining a great redundancy of constraints (homologous points connecting successive positions of the rig) while allowing for a large ratio between base distance and capturing distance, i.e., the ratio between the distance of the centres of gravity of two consecutive positions of the rig and the distance between the centre of gravity of the rig and the photographed surface, is equal to 1:1, while instead, using rectilinear projection optics, this ratio must be lower (about 1:2), significantly increasing the number of images required. Finally, the short focal length favours a wide depth of field that allows for one to simultaneously obtain very close and distant objects in focus, improving the result of the subsequent processing.

The device allows us to obtain a reconstruction directly to scale without the need for additional support measurements by exploiting the rigid, calibrated relative position of the cameras and the synchronization of the cameras.

This characteristic allows for the detection of a large number of key points in each direction around the multi-camera and a large number of image constraints (homologous points) between cameras within the structure itself and between consecutive positions of the structure during movement. The wide viewing angle means that the same key points can be recognized and used as constraints for many consecutive positions of the rig before they no longer fall within the cameras’ field of view. The redundancy of these constraints reduces drift error in prolonged acquisitions.

The instrument is designed to be held and used by a single operator walking independently through the environment/tunnel to be detected at normal walking speed and allow for a complete acquisition in a very short time. The acquisition proceeds in a completely autonomous way and can therefore be completed even by mounting the structure on a vehicle or other mode of movement without the need for the presence of the operator.

The key elements of the system are as follows:

- Global shutter cameras: The use of global shutter sensors allows for the reliable exploitation of the calibrated internal orientations.
- Circular fisheye lenses, with a field of view of 190°, arranged in a semicircle, allow for a hemispherical shot of the framed scene, excluding the operator, allowing for omnidirectional tie-point extraction. In addition, they provide a wide depth of field, allowing for the use of fixed focus while still covering close to faraway subjects.
- Rig geometry: The relative arrangement between the cameras favours determining the device’s position at the moment of acquisition and allows for omnidirectional constraint points that make the final reconstruction more robust.
- Calibrated RO: The constrained, rigid, and calibrated position between the cameras allows for automatic and accurate scaling, even in very large environments. The accurate hardware synchronization of the cameras guarantees consistent results.

The most notable advantages of the developed system are as follows:

- Contained drift error in prolonged acquisitions as evaluated on the field through challenging case studies [5,22,23,24,50].
- Reduced number of images required for 360° 3D reconstruction thanks to the wide viewing angle of the fisheye optics.
- Scaled reconstructions without additional support measures, thanks to the relative position of the calibrated cameras and their accurate synchronization.
- Speed and reliability of the acquisition, feasible even for non-photogrammetric experts.

### 4.2. Acquisition and Processing

The images are acquired with a time-based synchronized trigger that can be set at different frame rates. For most cases, during testing, a frame rate of 1 fps has been used. The image set acquired is stored in five subfolders, dividing the images according to the camera that acquired them. For the test performed so far, the processing step is performed using the Agisoft Metashape software (versions 1.7–2.0). Each camera is loaded with a reliable pre-calibration of the internal and relative orientation parameters. At this point, the image set can be oriented using the SfM implementation of Metashape.

Within the testing phase, it was observed that to achieve optimal results in challenging scenarios, such as complex and extensive interconnected tunnel environments, adjustments such as (i) “manual refinements” and (ii) “tie-points filtering” were necessary.

- Manual refinements: During the many tests performed, misalignments have been found multiple times. In the presence of misalignment, the approach used is that of a manual intervention to correct the mistakes. This is carried out by identifying the incorrectly oriented images, resetting them, and trying to re-align them. Trying to re-align a few images is usually successful since the software overwrites the valid and invalid matches selection which usually improves when most images are already oriented correctly. These operations have been implemented in the software Metashape using scripting so that by just selecting one of the images of the multi-camera pose, the re-alignment procedure is performed for all images of that multi-camera. With the experience gathered during the testing phase, the misalignment is progressively reduced to the point of mostly never needing to intervene in the initial orientation. The key to the improvements was that of increasing the redundancy in extremely complex areas by slowing down the walking speed. Complex areas include those characterized by a complex geometry, by poor texture, or by extreme contrast of illumination.
- Tie-point filtering: The removal of poor tie points is performed by exploiting the “gradual selection” tool available in the software Metashape that offers few metrics to select tie points to be removed. Through the investigation, the metrics “reprojection error” and the “reconstruction uncertainty” were used to remove up to around the 10% worst-performing tie points.

Ultimately, the adjustments slowed down the processing phase by requiring manual steps to be added to the process. The source of the misalignments can be identified in a too-complex image network, together with the lack of any effective strategy to pre-select the matching image pairs in large datasets. The problem can be solved by providing initial values for the exterior orientation of all images in the set, and this can be obtained through (i) a low-resolution pre-processing (still potentially requiring manual intervention) or (ii) real-time processing of the images during acquisition using visual SLAM algorithms [21]; the first experiments of the visual SLAM integration with Ant3D have been investigated in [49].

**Figure 17:**
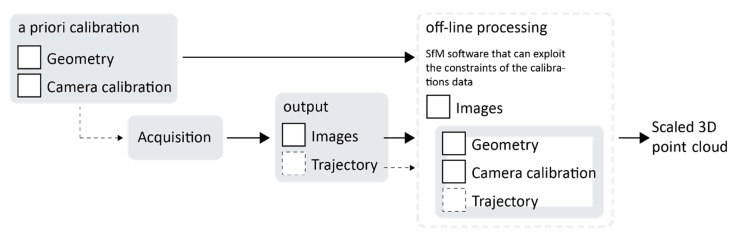
Scheme of processing phase.

Figure 17 shows a synthesis scheme of the processing phase. At the current implementation, the only output of the acquisition is the image dataset. However, in a future implementation, it is planned to integrate a real-time processing phase during acquisition that would also output the system trajectory based on the estimated image’s coordinates in object space.

### 4.3. Case Studies and Discussion

One of the case studies identified to test the instrument performance is the Castle of San Vigilio, the same subject of the acquisition of the FINE benchmark. The acquisition of this area allows us to verify the improvements made to the multi-camera system from its first definitions to that of the working prototype. The ground truth point cloud obtained with the laser scanner for the benchmark dataset was used as a reference survey. The best results obtained from the FINE benchmark data processing reported an error of more than 10 cm at the ends of the reconstruction up to errors of more than one metre for the worst reconstructions. In general, the processing of the first multi-camera acquisitions, although they have demonstrated the potential of the approach, has also highlighted the poor reliability and repeatability of the results caused by the limited hardware characteristics of the first iterations of the instrument.

Two major limitations were highlighted in the use of the GoPro action camera rolling shutter sensors and in the geometry of the system. The latter is characterized by cameras oriented mainly in the direction of the walk, which limits the possibility of tying the acquired photos in the forward direction with those acquired in the return direction. Carrying out this test allows us to assess the improvements to the new design.

A single acquisition was performed with the proposed device to acquire the whole area in a short time. The acquisition starts from the level of the tunnel, immediately below the access manhole. It proceeds along the underground tunnel, reaches the end, and returns to the starting point. Here, it continues up the manhole by means of a ladder and proceeds along the vaulted room with a central plan at the base of the tower. It then continues up the connecting stairs to the second level, surveys the vaulted room, and continues to the top with the last stretch of stairs. Outside, the acquisition runs along the external structure of the last stairwell and proceeds to acquire the external top of the tower. From here, it retraces its steps and retraces the path in the opposite direction back to the circular room on the ground floor; it then proceeds outside, surveying the outer surface of the tower. Finally, the acquisition returns to the circular room, which ends after a reinforcement connection through the access manhole to the tunnel. This time, the acquisition is carried out without the presence of the ladder.

**Figure 18:**
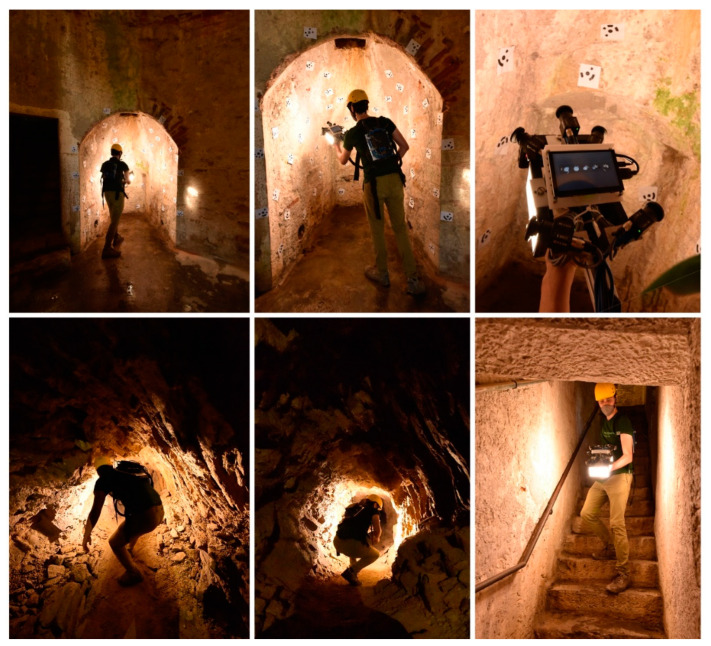
Images of the survey operation on the field, the acquisition of the self-calibration testified (first row), and the castle areas (second row).

**Figure 19:**
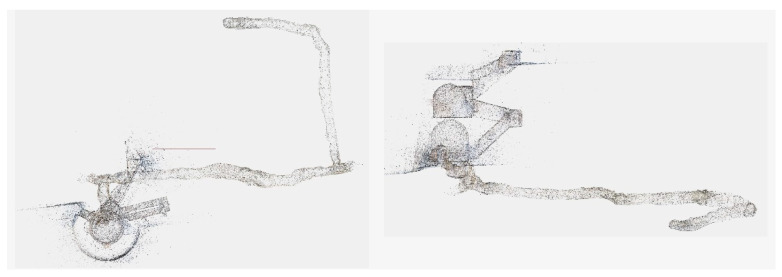
Plan view (**left**) and elevation view (**right**) of the San Vigilio reconstruction.

The steps performed during data processing are the ones described in Section 4.2; however, in this case, it was not required to perform any manual adjustment over the SfM output, and contrary to what was observed with the multi-camera data of the FINE benchmark, all the images were oriented correctly. In addition, it was observed that, thanks to the field of view of the circular fisheye optics used and the angle of the cameras with respect to the walking direction (directed more towards the walls of the tunnel), many more points of connection between the images taken from the outward and return path were detected. Figure 18 shows the acquisition phase of both the on-site calibration test field and the actual tower environments; Figure 19 shows the sparse point cloud.

For the final evaluation of the reconstruction, it was not possible to compare based on checkpoints as previously completed for the FINE benchmark acquisitions since the reference targets were removed. A direct comparison with the ground truth scanner point cloud was then carried out.

**Figure 20:**
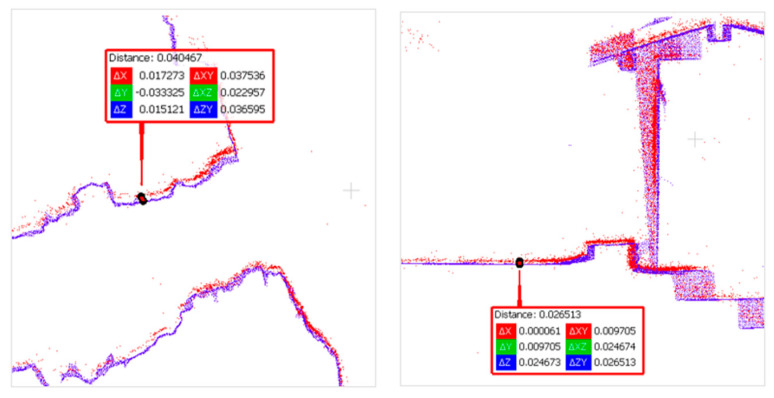
The distance between the multi-camera (red) and the reference (blue) point clouds on the left at the extreme of the tunnel (4 cm) and on the right at the extreme of the tower (2.6 cm).

**Figure 21:**
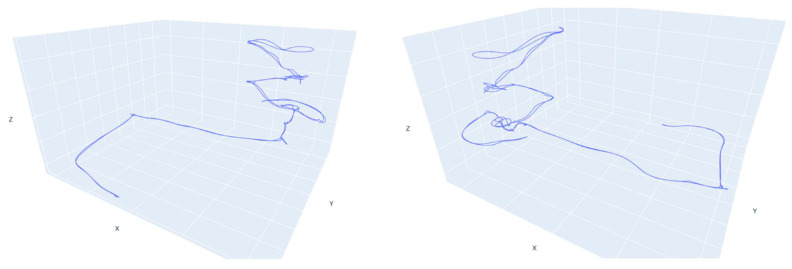
A plot of the trajectory followed during the San Vigilio survey.

The reference point cloud and the multi-camera reconstruction cloud were oriented by performing a best-fit registration on the surface of the circular access room to the tower. The two clouds were segmented and oriented using the Cloud Compare software (version 2.10). The point cloud transformation matrix, obtained from the best fit, was then applied to the complete point cloud of the multi-camera reconstruction, and the comparison between the two was made. Figure 20 shows some zoomed-in details of the overlapped point clouds (reference in blue, Ant in red). The comparison shows that the photogrammetric reconstruction is complete in all its parts and matches the laser scanner. The maximum deviation between the clouds, measured by performing sections in correspondence of the most extreme areas, was 4 cm at maximum, certifying the survey as suitable for the scale of architectural representation of 1:100. Figure 21 shows the survey trajectory followed during the acquisition.

**Figure 22:**
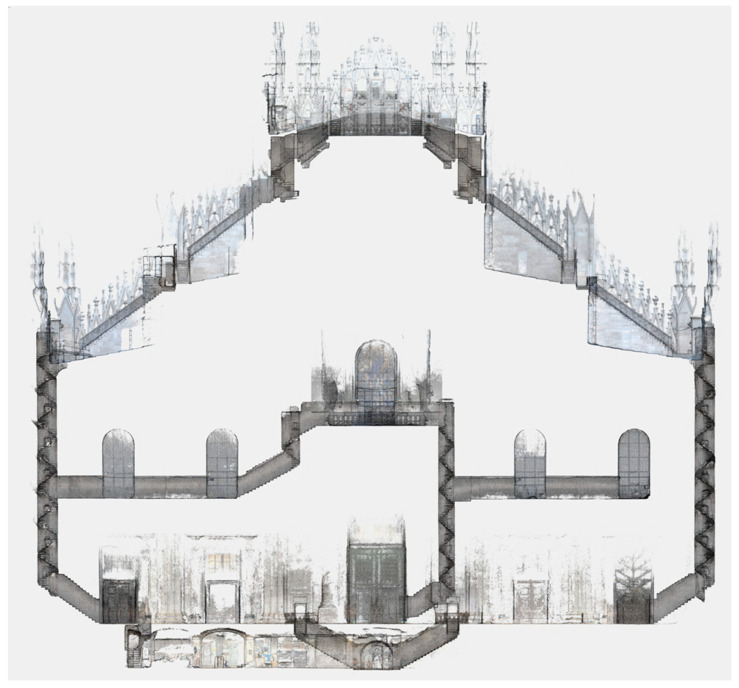
The elevation view of the point cloud generated from the Ant3D survey of Milan’s Cathedral façade’s narrow spaces and corridors. The image was extracted from a prior publication; additional details on the processing can be found at [22].

**Figure 23:**
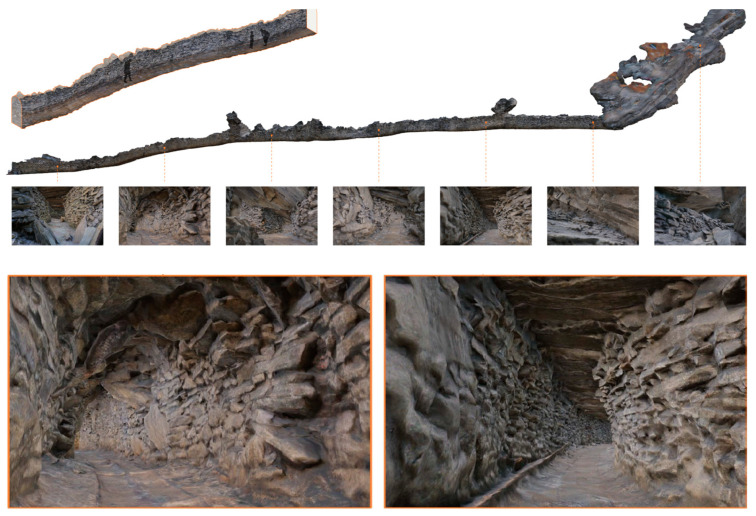
The 3D model generated from an Ant3D survey of an abandoned mining tunnel. Images extracted from a prior publication; additional details on the processing can be found at [24].

After the initial evaluation, the system was thoroughly put to the test further in similar scenarios to the one just presented, i.e., the main target application for the multi-camera, as well as for other types of applications not originally considered. Among the tests performed in the target application, most notably, Ant3D was used to complete the survey of the network of narrow spaces and staircases of Milan’s Cathedral (Figure 22), partially presented in [22]. Among the tests performed in other scenarios, there is (i) the acquisition of a mountain trail path [5], (ii) the acquisition of large [23] and narrow [24] mining tunnels (Figure 23), and (iii) the survey of a historical garden [50].

## 5. Conclusions and Future Works

This investigation aimed to develop an image-based measurement system capable of completing the three-dimensional digitization of complex and narrow spaces while acquiring high-resolution photographic documentation.

After an initial experimentation phase based on the use of low-cost hardware culminated in the FINE benchmark, numerous limitations were highlighted, such as (i) the suboptimal multi-camera acquisition geometry; (ii) the use of low-cost rolling shutter type sensors, not suitable for moving acquisitions; and (iii) the poor acquisition synchronization between the component cameras of the system, which limited the stability of the rigid distances between the cameras and thus the effectiveness of them as a constraint during the processing phase.

At this point, the objective was to overcome these limitations to arrive at the definition of the working prototype of the proposed multi-camera system. This phase saw the overcoming of the low-cost hardware used previously, favouring specialized sensors that could produce more accurate results, and removing the uncertainty introduced by the lack of synchronization between the cameras and rolling shutter sensors.

It has been verified that the hardware specifications of the working prototype (global shutter sensors and synchronization error lower than 200 microseconds) do not introduce measurable distortions in the conditions of intended use. In addition, an optimal acquisition geometry for the survey of confined indoor spaces has been defined using an approach based on the simulation of camera resolution as a function of the environment. The numerous tests carried out in the field [5,22,23,24,50] have demonstrated the effectiveness of the proposed solution that has allowed us to achieve the following objectives: to simplify and speed up the data acquisition phase and ensure an accuracy consistent with the architectural representation. All the selected case studies present a high level of difficulty and have different characteristics, covering a wide sample of possible real applications: from cultural heritage to archaeology and industry, from natural to artificial environments, and from extremely narrow spaces to larger tunnels and outdoor areas.

Moreover, the instrumental drift entity was controlled for all the case studies. The initial part of the executed path has been constrained. The error has been verified at the opposite end without the use of support measurements along the path’s extension. The results highlight the proposed solution’s robustness compared to existing alternatives on the market, such as portable, lightweight mobile range-based mobile mapping systems. The evaluation test presented confirmed the previous findings regarding the drift error robustness of the system. Constraining the survey only at the starting point, we measured an end drift error in the unconstrained survey path of 4 cm for a tunnel length of 80 m resulting in an estimated drift error for the proposed system of 5 cm per 100 m of unconstrained path. The results obtained are comparable to previous tests performed with the same system in more challenging scenarios: in [22], Ant3D was tested for the survey of meandering narrow passages in cultural heritage resulting in a drift error of around 5 cm per 100 m; in [23], for the survey of a 2 km long mining tunnel, the resulting drift error was around 9 cm per 100 m; and in [5], for the survey of a 3.5 km long mountain footpath, the resulting drift error was around 2 cm per 100 m. In the latter test, the drift error registered from a compared range-based MMSs was 20 cm per 100 m.

**Figure 24:**
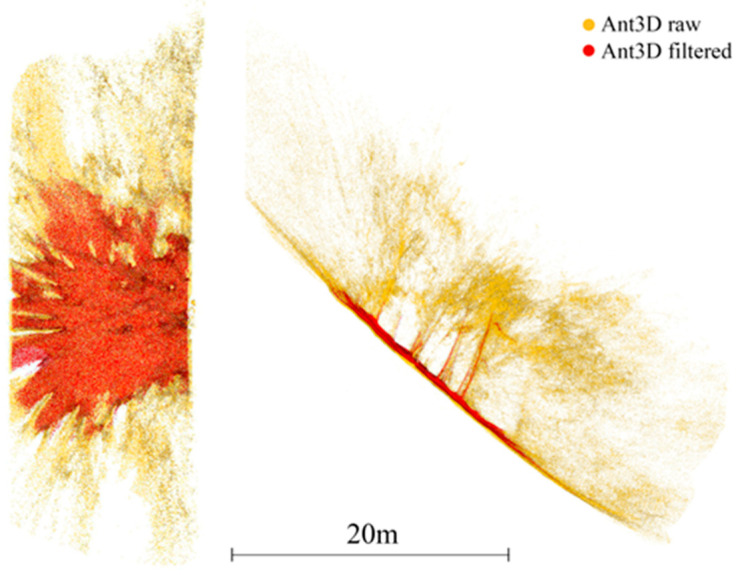
The plan and section view of an Ant3D point cloud acquired in a forest environment. In yellow is the full point cloud, and in red is a filtered point cloud limited to the quality data. Extracted from a prior publication [5].

**Figure 25:**
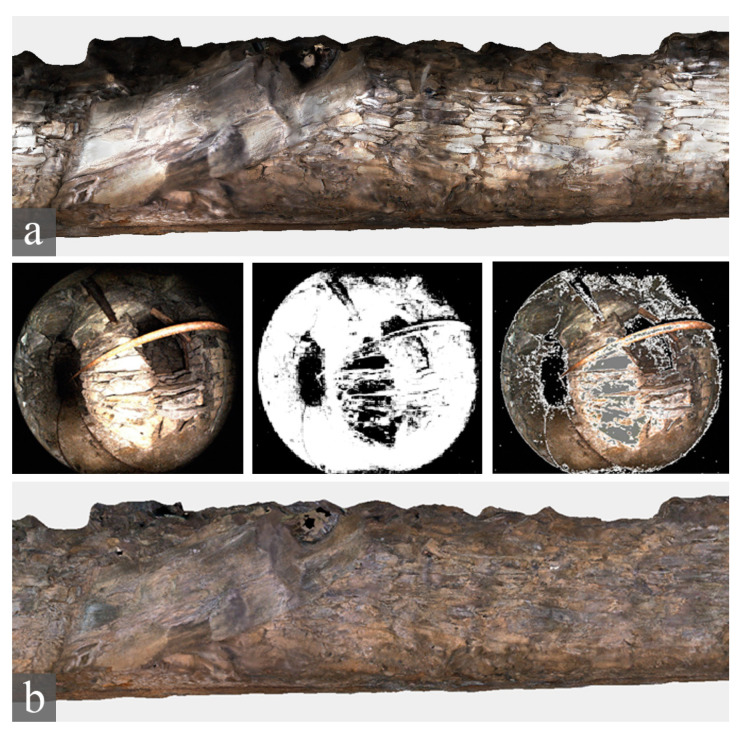
An orthomosaic generated from an Ant3D survey, from original image data (**a**), and from edited data masking highlights and dark shadows (**b**). Extracted from [24].

The current limitations of the system are (i) the limited range of the quality data generated around the system by multi-view stereo matching, (ii) the inhomogeneous or insufficient illumination of dark indoor areas, and (iii) the lack of real-time visual SLAM processing and acquisition feedback to the user. Regarding point (i), the point cloud derived from full resolution processing of the Ant3D images results in dense, complete, and accurate results in the limited range of around 5 m from the sensor which is suitable for the survey of narrow spaces yet limits the application of the system in outdoor environments as reported in [5,50] (Figure 24 extracted from [5]). Regarding point (ii), the illumination of narrow environments is limited by the goal of portability and manoeuvrability of the multi-camera that can only illuminate the environment from a single point. This results in inhomogeneous illumination the more the tunnel environments are narrow and the surfaces rough. So far, solutions have been explored in the post-processing phase by editing the images or output model texture (Figure 25 extracted by [24]). Moreover, the resolution of the cameras currently employed is limited: optimal to contain processing time, but also limited for high-resolution image documentation and lower than most 360° recording approaches. Regarding point (iii), for all case studies so far, Ant3D was always operated by an expert user who could perform a proper acquisition even without real-time processing feedback. Tests in the integration of visual SLAM processing are ongoing to both improve the usability for non-experts as well as the high-resolution post-processing of the data with initial EO estimates [49].

For future works, we envisage the integration of visual SLAM real-time processing as well as the integration of additional sensors such as multi-beam LiDAR and high-resolution rolling shutter cameras to be included just from model texturing and image documentation.

## 6. Patents

The work reported in this manuscript resulted in the patent proposal n° 102021000000812 for the ANT3D system, a novel multi-camera measuring device for surveying tunnels, mines, and generally narrow and complex spaces. The patent was licensed on 24 January 2023.

## Figures and Tables

**Table 1:** An evaluation of the different tests. The table reports the residuals on the CPs (black points) with the three reconstructions oriented over the GCPs (white points). The table refers to the FINE benchmark dataset.

		1 fps	2 fps	4 fps	CPs Scheme
n° tie points [M]		3.1	6.8	15.3	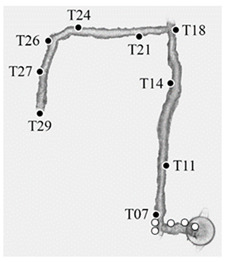
CPs [cm]	T07	1.92	1	15.99	
	T11	1.38	2.38	30.82	
	T14	2.2	2.78	55.39	
	T18	5.21	6.61	75.94	
	T21	3.55	4.59	75.65	
	T24	2.82	3.69	89.26	
	T26	4.01	4.28	89.36	
	T27	7.12	6.6	83.55	
	T29	13.34	10.9	75.34	
	worst	13.34	10.9	89.36	

## Data Availability

The original Fine benchmark data is available on request: please contact luca.perfetti@unibs.it, francesco.fassi@polimi.it or remondino@fbk.eu. For inquiries regarding the data presented in chapters 3, 4 and 5 please contact luca.perfetti@unibs.it.
